# Damaged fiber tracts of the nucleus basalis of Meynert in Parkinson’s disease patients with visual hallucinations

**DOI:** 10.1038/s41598-017-10146-y

**Published:** 2017-08-31

**Authors:** Dagmar H. Hepp, Elisabeth M. J. Foncke, Henk W. Berendse, Thomas M. Wassenaar, Kim T. E. Olde Dubbelink, Henk J. Groenewegen, Wilma D.J. van de Berg, Menno M. Schoonheim

**Affiliations:** 1 Department of Neurology, Neuroscience Campus Amsterdam, VU University Medical Center, Amsterdam, The Netherlands; 2 Department of Anatomy and Neurosciences, Neuroscience Campus Amsterdam, VU University Medical Center, Amsterdam, The Netherlands

## Abstract

Damage to fiber tracts connecting the nucleus basalis of Meynert (NBM) to the cerebral cortex may underlie the development of visual hallucinations (VH) in Parkinson’s disease (PD), possibly due to a loss of cholinergic innervation. This was investigated by comparing structural connectivity of the NBM using diffusion tensor imaging in 15 PD patients with VH (PD + VH), 40 PD patients without VH (PD − VH), and 15 age- and gender-matched controls. Fractional anisotropy (FA) and mean diffusivity (MD) of pathways connecting the NBM to the whole cerebral cortex and of regional NBM fiber tracts were compared between groups. In PD + VH patients, compared to controls, higher MD values were observed in the pathways connecting the NBM to the cerebral cortex, while FA values were normal. Regional analysis demonstrated a higher MD of parietal (*p = *0.011) and occipital tracts (*p = *0.027) in PD + VH, compared to PD − VH patients. We suggest that loss of structural connectivity between the NBM and posterior brain regions may contribute to the etiology of VH in PD. Future studies are needed to determine whether these findings could represent a sensitive marker for the hypothesized cholinergic deficit in PD + VH patients.

## Introduction

Visual hallucinations (VH) in Parkinson’s disease (PD) are common and reflect a more malignant disease course, including a high risk of dementia^1^. Although the pathophysiology of VH in PD is still poorly understood, a cholinergic deficit is thought to be an important factor^2, 3^. According to the hypocholinergic hypothesis of VH in PD, decreased cholinergic innervation of the visual association cortex may lead to an inability to suppress intrinsic cortical activity during perception, and thereby to VH^4^. This hypothesis is supported by the tendency of anticholinergic drugs to induce VH^4, 5^. Conversely, cholinesterase inhibitors have been shown to ameliorate VH in PD patients, in addition to their beneficial effect on cognitive function, although the therapeutic response is variable^6–8^.

The nucleus basalis of Meynert (NBM) is located in the basal forebrain, namely in the subcomissural area traditionally indicated as substantia innominata (SI)^9^. The NBM constitutes the primary source of cholinergic innervation to the cerebral cortex^10, 11^ and, in neuropathological studies, severe neuronal loss within this nucleus has been reported in PD^12, 13^. In previous volumetric magnetic resonance imaging (MRI) studies, atrophy of the SI was reported in PD patients with VH (PD + VH), compared to PD patients without VH (PD − VH) and SI atrophy was associated with cognitive decline^14, 15^.

As the NBM degenerates, it is likely that the fiber tracts connecting the NBM with cerebral cortical brain areas will also be affected. Demonstrating a disruption of these fiber tracts would not only further support the hypocholinergic hypothesis of VH in PD, but could also provide us with an *in vivo* measure of the severity of the cholinergic deficit in PD. Diffusion tensor imaging (DTI) is a brain imaging method that can be used to investigate the integrity of fiber tracts in the human brain^16^. DTI may therefore provide additional information over structural MRI in characterizing the cholinergic deficit in PD. In Alzheimer’s disease, another neurodegenerative disease with a major cholinergic deficit, atrophy of the SI correlated with decreased integrity of intracortical projecting fiber tracts^17^. In PD, only a few previous studies used DTI to compare fiber tract and/or tissue integrity in patients with and without VH, but none of these included the NBM as region of interest^18–20^.

In the present study, we aimed to investigate whether the integrity of connections between the NBM and cerebral cortical regions is disturbed in PD + VH, compared to PD − VH patients and controls, by performing seed-based probabilistic tractography using DTI. We expected a loss of integrity of fiber tracts between the NBM and cerebral cortex in PD + VH patients, compared to PD − VH patients. In addition, we explored whether changes in structural connectivity of the NBM would be associated with cognitive impairments.

## Results

### Demographic and clinical data

Gender distribution, age and educational level were comparable between PD patients and controls (see Table 1). Global cognitive functioning, as measured with CAMCOG, was lower in PD patients compared to controls (*U = *194, *p = *0.004), whereas MMSE scores were comparable (*U = *362, *p = *0.721). Comparing PD + VH and PD − VH patients, disease duration and disease stage were similar in both groups (*t*(53) = −0.61, *p = *0.547 and Fisher Exact *p = *0.550, respectively), but motor performance (UPDRS III; *t*(53) = −2.26, *p = *0.028) and global cognitive functioning (CAMCOG; *U = *165; *p = *0.011) were worse in PD + VH patients. The LEDD and the use of dopamine agonists, neuroleptics, amantadine and MAO-B inhibitors were comparable between patient groups. Four PD − VH and two PD + VH patients used medication with an anticholinergic effect (Fisher Exact, *p = *0.660) and no PD − VH, but five PD + VH patients used a cholinesterase inhibitor (rivastigmine; Fisher Exact, *p = *0.001).

**Table 1:** Demographics and clinical measures of PD patient groups and controls.

	Controls	PD − VH	PD + VH
Nr. of subjects	15	40	15
Male nr. (%)	10 (67)	21 (53)	11 (73)
Age mean years ± SD	67 ± 8	67 ± 7	69 ± 4
ISCED (0/1/2/3/4/5/6)	0/ 0/ 2/ 3/ 1/ 8/ 1	0/ 0/ 14/ 11/ 2/ 13/ 0	0/ 0/ 4/ 4/ 0/ 6/ 1
Disease duration mean years ± SD	n/a	11 ± 4	12 ± 4
UPDRS III mean score ± SD	n/a	30 ± 10	37 ± 9^#^
H&Y (2/ 2.5/ 3)	n/a	16/ 16/ 8	6/ 4/ 5
MMSE score ± SD	28 ± 1	28 ± 1	26 ± 4
CAMCOG score ± SD	99 ± 3*	95 ± 7	87 ± 11^#^
LEDD mean dose mg ± SD	n/a	1008 ± 610	1081 ± 446

Legend Abbreviations: PD = Parkinson’s disease, VH = Visual Hallucinations, ISCED = International Standard Classification of Education, UPDRS III = Unified Parkinson’s Disease Rating Scale part III, H&Y = Hoehn and Yahr stage, MMSE = Mini Mental State Examination, CAMCOG = Cambridge Cognitive Examination-Revised test battery, LEDD = Levodopa Equivalent Daily Dose.

*p < 0.05 compared to PD (PD + VH and PD − VH).

^#^ *p < *0.05 com*p*ared to PD − VH.

### NBM volume and whole-brain connectivity

**Figure 1:**
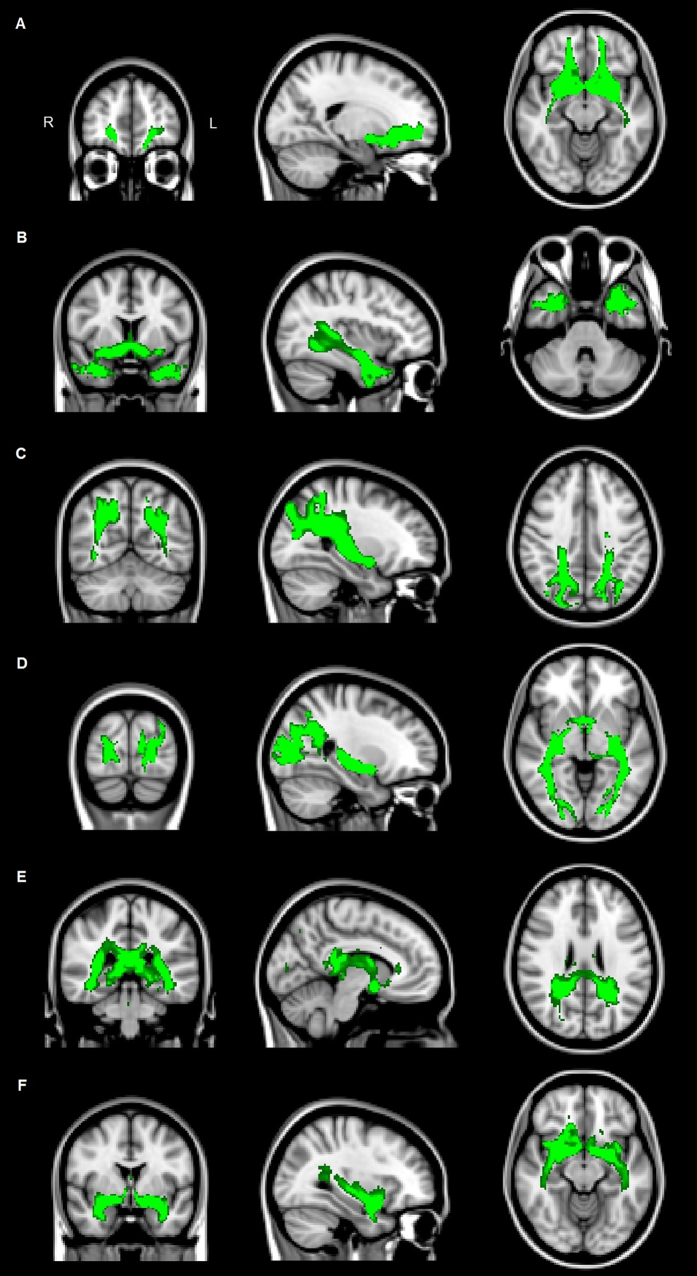
Tracts between the NBM and cortical regions in controls. Legend Regional tracts between the NBM and frontal (**A**), temporal (**B**), parietal (**C**), occipital (**D**) and cingular cortex (**E**), and insula (**F**) in controls. Abbreviations: NBM = nucleus basalis of Meynert.

The normalized volume of the NBM was comparable in PD + VH, PD − VH patients and controls (*p = *0.368). Normalized total brain volumes were also comparable between groups (*p = *0.837). The FA of the tracts between the NBM and the rest of the brain was comparable between PD + VH, PD − VH and controls (*p = *0.402) and was not investigated regionally. MD values were significantly different between groups, i.e. highest in PD + VH patients and lowest in controls (*p = *0.049). Post-hoc comparisons between groups demonstrated only a significantly higher MD in PD + VH, compared to controls (*p = *0.043), and an absence of significant differences in MD between PD − VH patients and controls (*p = *0.428), and between PD + VH and PD − VH patients (*p = *0.404). The six regions showing the strongest connectivity with the NBM in controls were the orbital part of the superior frontal gyrus, the middle temporal gyrus at the temporal pole, the superior parietal gyrus, the superior occipital gyrus, the posterior cingulate gyrus and the insula (Fig. 1).

### Regional tracts: group comparisons and relation with clinical parameters

**Figure 2:**
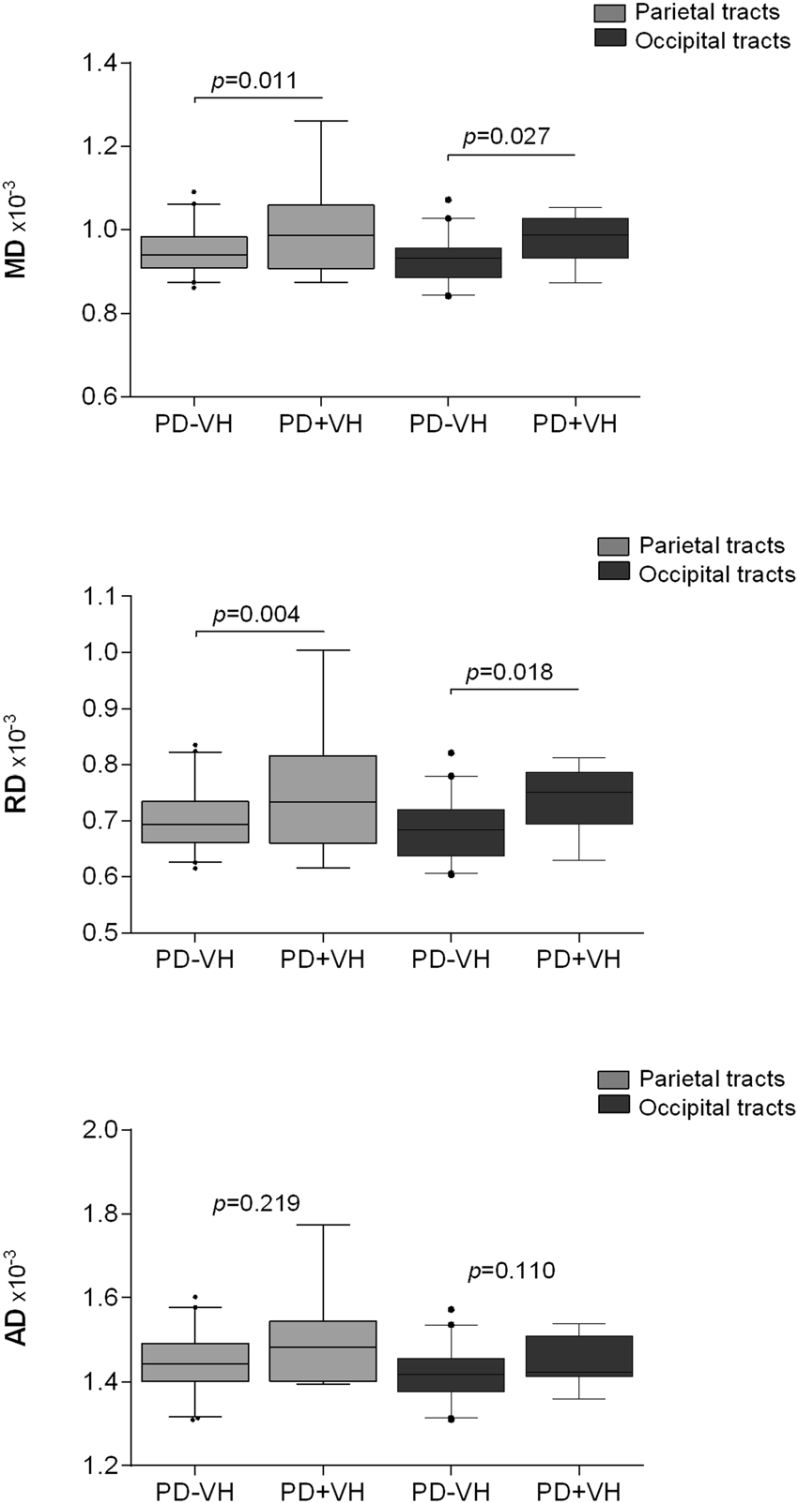
Posterior cortical NBM denervation in PD + VH patients. Legend The MD of parietal and occipital tracts was higher in PD + VH, compared to PD − VH patients (upper panel, *p*-values shown are corrected for gender, age, education and multiple group comparisons). Post-hoc comparisons showed that diffusivity differences were mainly driven by differences in RD (middle panel) and not AD (lower panel). Box displays first to third quartile and median (line), and whiskers display 5–95 percentile. Abbreviations: MD = Mean diffusivity, RD = Radial diffusivity, AD = Axial diffusivity, PD = Parkinson’s disease, VH = Visual Hallucinations, NBM = nucleus basalis of Meynert.

Investigating the MD of tracts between the NBM and six most strongly connected brain regions in more detail, the MD of parietal (*p = *0.013) and occipital tracts (*p = *0.026) was different between all three groups (Table 2). Post-hoc comparisons showed a significantly higher MD of parietal and occipital tracts in PD + VH, compared to PD − VH patients (*p = *0.011 and *p = *0.027, respectively). Only for the parietal and occipital tracts were AD and RD values explored, showing a higher RD in PD + VH, compared to PD − VH patients (parietal tracts: *p = *0.004; occipital tracts: *p = *0.018; Fig. 2), while AD values were comparable between both patient groups (parietal tracts: *p = *0.219; occipital tracts: *p = *0.110). There was a trend towards a correlation between a higher MD of the parietal tracts and worse total CAMCOG scores in PD patients (*rho = *−0.226, *p = *0.097), and no relation between MD of parietal and occipital tracts and general measures of disease progression, i.e. disease duration or motor performance.

**Table 2:** Tracts between the NBM and different cerebral cortical regions

	Controls	PD − VH	PD + VH	*p* main effect	*p* HC vs PD − VH	*p* HC vs PD + VH	*p* PD − VH vs PD + VH
**Frontal** tracts MD (×10^–3^ mm^3^)	1.03 ± 0.06	1.04 ± 0.08	1.09 ± 0.10	0.156	na	na	na
**Temporal** tracts MD (×10^−3^ mm^3^)	1.01 ± 0.07	1.04 ± 0.06	1.06 ± 0.08	0.138	na	na	na
**Parietal** tracts MD (×10^−3^ mm^3^)	0.93 ± 0.04	0.93 ± 0.05	0.97 ± 0.06	**0.013**	1.000	0.118	**0.011**
**Occipital** tracts MD (×10^−3^ mm^3^)	0.95 ± 0.05	0.95 ± 0.05	1.00 ± 0.10	**0.026**	1.000	0.113	**0.027**
**Cingular** tracts MD (×10^−3^ mm^3^)	1.05 ± 0.07	1.04 ± 0.07	1.07 ± 0.11	0.404	na	na	na
**Insular** tractsMD (×10^−3^ mm^3^)	1.03 ± 0.08	1.06 ± 0.09	1.08 ± 0.08	0.480	na	na	na

Legend All MD-values are given in mean ± standard deviation. *p*-values shown are corrected for gender, age and education and post-hoc comparisons between groups are corrected for multiple comparisons (Bonferroni correction). Significant *p*-values are shown in bold. Abbreviations: MD = Mean diffusivity, PD = Parkinson’s disease, VH = Visual Hallucinations, na = not applicable.

## Discussion

We investigated the integrity of NBM tracts in PD patients with and without VH and controls and report reduced tract integrity, indicated by a higher MD in PD + VH patients, compared to controls. Regional analyses demonstrated a higher MD of parietal and occipital tracts in PD + VH, compared to PD − VH patients. This effect of MD appears to be driven by an increase in RD, not AD. The volume of the NBM was not significantly different between groups. This indicates that reduced fiber integrity of parietal and occipital NBM tracts may be relevant to the etiology of VH in PD and suggests that tractography may be a sensitive marker for the cholinergic deficit in PD + VH patients.

A higher MD, driven by higher RD, in the tracts between the NBM and posterior cortical brain regions in PD + VH patients may reflect the presence of microstructural damage of these tracts. To our knowledge, the state of myelination of the cholinergic fibers originating in the NBM has not been studied in humans. In animal studies, the ascending cholinergic projections from the basal forebrain are mostly unmyelinated^11, 21^. Considering that myelination is an important factor in DTI analyses, one might not expect DTI to be sensitive to damage to largely unmyelinated axons of NBM projections. However, lesioning of the basal forebrain in an experimental mouse model revealed that lesioned animals had lower tract integrity as measured with DTI^22^. Interestingly, as in the present study in humans, only increases in MD and RD were reported in the tracts of lesioned animals, while the FA and AD remained normal. Apparently, degeneration of NBM neurons and axons may lead to debris and/or changes to axonal organization and packing, which may particularly influence MD and RD^22^. Future studies using more advanced DTI protocols with higher resolutions and more diffusion directions, as well as techniques like neurite orientation dispersion and density imaging (NODDI), may provide more insights into the type of microstructural changes of NBM connections in PD + VH patients^23^.

With regard to the predominant involvement of parietal and occipital fiber tracts in our PD + VH patients, it is interesting to note the topographical organization of the NBM. The NBM can be broadly subdivided into three divisions (anterior, intermediate and posterior) whereby each division innervates specific cortical regions, i.e. the anterior division innervates frontal, limbic and medial cortical regions, the intermediate division innervates parietal and occipital regions, and the posterior division innervates the superior temporal gyrus and temporal pole^10, 24^. As our results show a predominant difference in parietal and occipital tracts, more severe PD-related pathology and/or neuronal loss in the intermediate NBM-division may occur in PD + VH patients, as previously suggested by others based on neuropathological observations^24^. In an *in vivo* tracer study, a fronto-occipital gradient of increasing cholinergic dysfunction was demonstrated in demented PD patients, also emphasizing the idea of more severe effect on the posterior cholinergic tracts in PD patients^25^. However, we were unable to include the entire anterior and posterior divisions of the NBM, since the most anterior and posterior parts cannot be reliably distinguished from the basal ganglia on MRI^10, 26^. Group effects in frontal, limbic, medial cortical and temporal NBM tracts may therefore be underestimated.

Our findings provide further evidence for the involvement of the NBM in the pathophysiology of VH in PD, by demonstrating NBM denervation in PD + VH, compared to PD − VH patients. Damage to the NBM tracts could result in decreased cholinergic innervation of visual associative cortical regions, including parietal and occipital regions, which may lead to the release of irrelevant and intrinsic sensory information into conscious awareness and thereby to VH^4^. In fact, cholinergic denervation has been implicated in multiple integrative models trying to explain VH in PD^27^. The ‘*Activation, Input, Modulation (AIM) model*’ has been proposed to explain VH in PD. In this model, disturbed rapid eye movement dreaming and vigilance abnormalities, caused by dysregulation of cholinergic brainstem centers, as well as aberrant functioning of associative frontal and occipital cortices, caused by deficits in cortical acetylcholine, are hypothesized to be important contributing elements^28^. In the ‘*Perception and Attention deficit (PAD) model’*, impairments in both attention (involving prefrontal cortex) and perception (involving occipital cortex) are believed to cause VH in PD, whereby cholinergic pathology is suggested to lead to disrupted communication between these brain regions^29^. In this model, so-called ‘proto-objects’ are envisioned as abstract object representations in competition to reach awareness by directing attention towards themselves. VH arise when the wrong proto-object is drawn into the attentional focus of a scene, which can occur because of an interaction between attentional binding and limited sensory activation of the correct proto-object^29^.

More recently, a hypothetical framework was postulated with a main focus on ‘*dysfunction of attentional control networks’* ^30^. In this framework, disturbed interaction between the dorsal attention network (DAN), the ventral attention network (VAN) and default mode network (DMN) is believed to cause VH. Normally, the DAN is important to focus attention on externally driven percepts. In PD + VH patients, the DAN is thought to be abnormally dormant, which is believed to induce an overreliance on the VAN and DMN, which are normally only involved in rapid reorienting of attention towards salient stimuli and retrieval and manipulation of episodic memories and semantic knowledge, respectively. As such, a global whole-brain attentional network imbalance ensues which may result in VH. This whole-brain attention network imbalance may strongly rely on a cholinergic imbalance, as cholinergic function is crucial for maintaining a normal level of selective attention^2^.

Across all these models, the NBM could play an important role, given the central role for a cholinergic denervation. Firstly, a cholinergic denervation as seen in PD has been associated with disturbed sleep and vigilance, both of which play an important role in the AIM model of VH in PD^31^. Second, the importance of acetylcholine-dependent attention is strongly emphasized in both the PAD model, as well as the model of ‘*dysfunctional attentional control networks*’ for the pathophysiology of VH. In the present study, PD + VH patients displayed reduced integrity of projections between the NBM (the primary source of acetylcholine in the brain) and parietal and occipital regions, which are implicated in the mechanistic models described above, and hence underline the theory that cholinergic denervation is a contributory factor in the pathophysiology of VH in PD.

In a previous study using transcranial magnetic stimulation, a reduced short-latency afferent inhibition (SAI) – which is believed to represent central cholinergic activity – was demonstrated in PD + VH, compared to PD − VH patients, also supporting the importance of altered cholinergic neurotransmission in the etiology of VH^32^. It is important to note here that in addition to the degeneration of the NBM, neuronal loss in the pedunculopontine nucleus in PD may contribute to the cortical cholinergic deficit in PD – and possibly the development of VH – as well, either via disturbed projections to the NBM or via reduced activation of thalamocortical projections^33^. Unfortunately, the location and size of the pedunculopontine nucleus prevented analysis hereof in the present study.

We did not observe differences in the NBM volume between PD + VH and PD − VH patients, nor between PD patients and controls, unlike previous reports^14, 15^. Clinical differences in PD patient cohorts may be an explanation for this disagreement, since our PD patients were less affected in terms of cognitive impairment. Interestingly, in the study comparing SI volumes in PD + VH and PD − VH patients, cognitive impairment in PD patients was quite severe (mean MMSE score = 25), while the mean disease duration was very short (only 3 months)^14^. Thus, in that study, patients with clinical phenotypes more closely related to dementia with Lewy bodies may perhaps have been included. Based on our findings, one may speculate that DTI is a more sensitive marker for the cholinergic deficit in PD + VH patients with a less progressive disease course regarding cognitive impairments. We did observe a trend for a correlation between the loss of tract integrity of parietal tracts and worse cognitive function, in line with previous work linking cholinergic denervation and cognitive decline in PD^25, 34^.

Normalized brain volumes were comparable between groups, but we did not investigate regional grey matter atrophy in the present study. Interestingly, atrophy of parietal and occipital brain regions in PD + VH, compared to PD − VH patients, has been reported in previous volumetric MRI studies^35–37^. Combining the results of these studies with ours, one may speculate that neuronal loss and pathology of the NBM may cause denervation of parietal and occipital brain regions, and, ultimately, atrophy of these regions. It should be noted, however, that the exact localization of the observed atrophy in PD + VH, compared to PD − VH patients, has been rather variable in previous reports, and increased volumes, as well as similar grey matter volumes have been reported as well, even when functional changes were already visible^37–40^. Thus, future studies are warranted to investigate the occurrence of regional atrophy in PD + VH patients, as well as its relation to connectivity changes, in more detail.

Interestingly, the NBM is currently under investigation as a target for deep-brain stimulation (DBS) in Alzheimer’s disease, and pilot data show stable or even improved cognitive function in 4 out of 6 patients treated with NBM-DBS^41^. The exact mechanisms underlying the therapeutic effect of NBM-DBS are still unclear, but increased neuronal firing of the NBM may provide a steady cholinergic neocortical background activity and thereby modulate the influences of other afferents to the neocortex. A case report of NBM-DBS in a demented PD patient revealed a remarkable improvement of cognitive functions associated with VH^42^. These and the present results provide further rationale for the investigation of NBM-DBS therapy for the treatment of VH and cognitive impairment in PD patients.

There are some limitations of the present study that ought to be mentioned. First, our study has a limited sample size and therefore, the results should be interpreted with caution. However, DTI studies in PD patients with and without VH are rare and sample sizes in these studies are comparable to ours^18, 19^. Second, (dis)integrity of non-cholinergic fiber bundles probably influenced our results, since the fiber tracts as identified by our method (Fig. 1) do not exactly resemble the cholinergic fiber tracts identified in a previous histopathological study^11^, and using the current method we cannot measure whether the tracts identified are cholinergic. Possible explanations for this dissimilarity include the intermingling of cholinergic pathways with non-cholinergic fiber bundles such as the uncinate fasciculus and the interference of non-cholinergic crossing fibers in the probabilistic tractography. Studies using DTI protocols with higher resolutions or techniques such as NODDI are needed to overcome this important limitation, in addition to post-mortem studies where cholinergic fibers can perhaps be identified directly. Third, five PD + VH patients and no PD − VH patients used cholinesterase inhibitors, which could have influenced our findings. In a DTI study in Alzheimer’s disease patients, the use of cholinesterase inhibitors temporarily normalized FA values^43^. It is possible, therefore, that the FA values were normalized in our PD + VH patients due to the use of cholinesterase inhibitors, warranting further study. Lastly, to reduce multiple-testing issues, we limited our regional tract analysis to six cortical target-regions. Group differences in tracts between the NBM and other cortical regions may have been missed due to this approach and future studies with larger sample sizes are needed to investigate this.

In summary, we provide evidence for damage to NBM tracts in PD patients with VH, and suggest that this may contribute to the pathophysiology of VH in PD through cholinergic denervation. We did not observe NBM atrophy in PD + VH patients and speculate that DTI might be a more sensitive marker for the cholinergic deficit in PD + VH patients. Future studies are needed to confirm whether posterior cholinergic denervation can predict the development of VH in PD. Ultimately, we hope that our findings may lead to better individualized and earlier treatment strategies in PD patients that are at risk for VH.

## Methods

### Subjects

All subjects were part of an existing longitudinal study cohort of *de novo* to moderately advanced idiopathic PD patients and healthy controls, of which the recruitment and inclusion specifics were reported previously^44, 45^. Briefly, PD patients were recruited from the outpatient clinic for movement disorders at the VU University Medical Center (VUMC) and a group of healthy controls was composed of spouses of the patients as well as other healthy volunteers. DTI registrations were performed at the first and second follow-up visits, 4 and 7 years after initial inclusion. Based on the availability of structural MRI and DTI scans and clinical data, we selected 55 PD patients (mean disease duration ± SD: 11 ± 3.7 years) and 15 age- and gender-matched controls for the present study. All PD patients fulfilled the clinical diagnostic criteria of the UK PD Brain Bank^46^. Participants gave written informed consent to participate in the research protocol, which was approved by the institutional ethics review board of the VU University Medical Center, in adherence to the Helsinki declaration.

### Clinical assessment

The presence of visual hallucinations (VH) in PD patients was assessed using the Scales for Outcomes in Parkinson’s disease-Psychiatric Complications (SCOPA-PC), which is a validated and easy-to-administer questionnaire for neuropsychiatric complications in PD^47^. Patients were classified as PD with or without VH (PD + VH or PD − VH) based on a score of either ≥ 1 or 0 on the first item of the questionnaire. None of the PD patients experienced isolated hallucinations in another modality (e.g. auditory, tactile and/or olfactory hallucinations). Disease duration was determined based on the time since the occurrence of the first motor symptoms as reported by the patient. The severity of motor symptoms was rated in the “ON” medication state using the Unified Parkinson’s Disease Rating Scale, part III (UPDRS-III)^48^ by a trained physician. A disease stage was attributed to each patient according to Hoehn and Yahr^49^. Cognitive function was assessed using the mini-mental state examination (MMSE) and the Cambridge Cognitive Examination-Revised test battery (CAMCOG-R), whereby a total CAMCOG-R score below 80 was considered indicative of dementia (max. total score = 105)^50^. The level of education was classified according to the International Standard Classification of Education (ISCED, UNESCO 1997), which ranges from 0 (no primary education) to 6 (university). The levodopa equivalent daily dose (LEDD) was calculated for each patient as described elsewhere^44^ and the use of cholinesterase inhibitors and other relevant medication (i.e. anticholinergics, dopamine agonists, neuroleptics, amantadine and MAO-B inhibitors) was registered.

### Image acquisition

All subjects underwent structural whole-brain 3T-MR scans (GE Signa HDXT, V15M), with a sagittal 3DT1 fast spoiled gradient-echo sequence (FSPGR, TR 7.8, TE 3.0, TI 450 ms, FA 12, 1.0 × 0.9 × 0.9 mm voxel size). Patients and controls with significant cerebrovascular lesions (Fazekas score > 1^51^) were excluded. Scanning was performed in the “ON” medication state. DTI acquisition consisted of five volumes without directional weighting and 30 volumes with non-collinear gradient directions (b-value 1000 s/mm^2^), repetition time 13275.0 ms; echo time 91 ms; 48 contiguous axial slices; slice thickness 2.4 mm; in-plane resolution 2 × 2 mm. All processing steps were performed using FMRIB’s Software Library (FSL) version 5.0 (http://www.fmrib.ox.ac.uk.fsl).

### Delineation of the nucleus basalis of Meynert (NBM)

**Figure 3:**
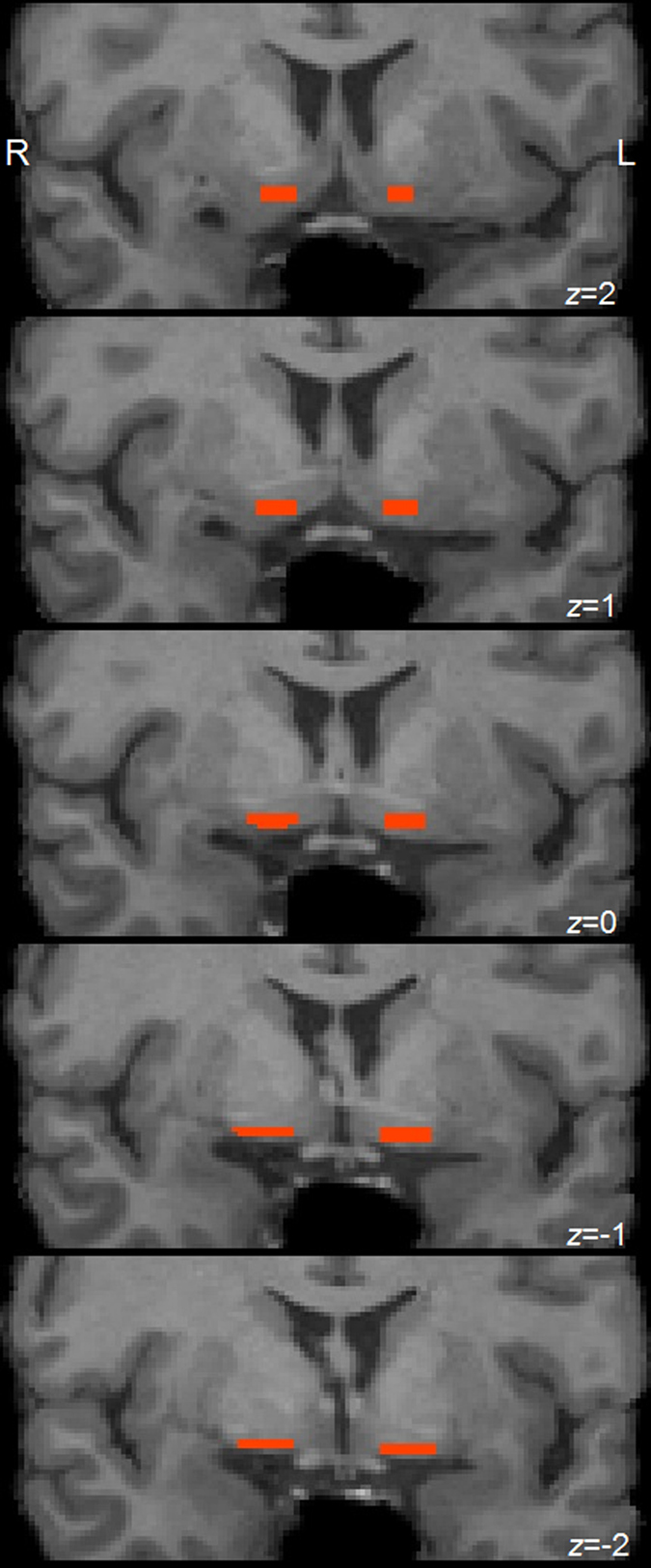
Delineation of the NBM in a healthy control. Legend Coronal sections from anterior (upper panel) to posterior (lower panel) encompassing the delineated NBM in a healthy control (red). Delineation was performed on 5 consecutive gapless 1 mm sections, of which the first was identified at the level of the anterior commissure (*z* = 0, third panel). Two more anterior and two more posterior sections were subsequently included. Abbreviations: NBM = nucleus basalis of Meynert.

Delineation of the NBM was based on a method described earlier^15, 52^, and was performed manually on T1-weighted 1.0 mm thick coronal slices reformatted to be perpendicular to the anterior commissure-posterior commissure axis by alignment with a standard brain, using an affine rigid-body registration. The NBM was outlined on five consecutive gapless 1 mm sections, of which the first was identified at the level of the crossing of the anterior commissure (AC), after which two more anterior and two more posterior sections were included (Fig. 3). The dorsal border of the NBM was defined as the most ventral aspect of the globus pallidus, while the ventral border was formed by the base of the brain. The lateral border of the NBM was demarcated by the medial aspect of the putamen, while the medial border followed the line extending from the ventrolateral border of the bed nucleus of the stria terminalis to the base of the brain. The anterior commissure was excluded from the ROI by keeping a minimum of one voxel between the anterior commissure and ROI during delineation. All tracings were carried out by DH, who was blinded to diagnosis and presence or absence of VH at the time of tracing, and were highly reproducible (intraclass correlation coefficient 0.95 between two repeated measurements in 10 cases). To calculate total normalized NBM volume, the NBM volumes of both hemispheres were added and multiplied by the V-scaling factor of SIENAX, i.e. the scaling factor between the individual and template skull. Total brain volumes (normalized for head size) were calculated as well using SIENAX.

### DTI analysis

All DTI images and gradient-vectors were corrected for movement and eddy current distortions using FMRIB Diffusion Toolbox (FDT, part of FSL). The NBM ROIs were registered to each individual DTI sequence using an inverted boundary-based registration (BBR) matrix. Subsequently, we followed a two-step approach to investigate the integrity of tracts between the NBM and individual cortical regions. Firstly, we investigated the integrity of the thresholded whole-brain connectivity map of the NBM between groups. Subsequently, we regionally explored individual tracts for those diffusion metrics that showed a significant whole-brain effect, but only in the most strongly connected NBM tracts per brain lobe, to reduce noise and the number of tests. Probabilistic tractography was applied using ProbTrackX, using standard settings and 5000 fibers, to localize the tracts connecting each individual NBM to the rest of the brain. We disregarded the lowest 0.02% of the total possible number of NBM fibers of the individual probabilistic maps, in line with previous imaging work^53^. The thresholded connectivity map of the NBM was binarized and used to calculate the mean fractional anisotropy (FA) and mean diffusivity (MD) of the total NBM tract. Cortical regions were based on the automated anatomic labeling (AAL) atlas, which was registered to each individual 3DT1 scan by inverting non-linear registration parameters, and subsequently to DTI space using an inverted BBR registration. Hereafter, a restricted structural connectivity analysis was performed between the NBM and each AAL region in controls, apart from the gyrus rectus and olfactory gyrus because of the magnetic susceptibility artifacts and distortions in these regions. Within each brain lobule, as well as in the cingulate gyrus and insula, only the most strongly connected AAL region was considered for further analysis.

### Regional NBM tracts: comparisons between groups

In the subsequent tractography analysis in each subject, each region was entered as a way-point as well as an end-point, in order to provide a map of all white-matter voxels included in the tract. These maps were binarized and used to determine the individual mean diffusion metrics for each tract. FA and/or MD were only explored in case of a significant main effect between the three groups. In case of a significant effect of MD, axial and radial diffusivities (AD and RD, respectively) were explored.

### Statistical analyses

We performed a Fisher exact test to evaluate categorical values across groups and evaluated continuous variables between groups with an independent t-test or ANOVA in the case of normally distributed data and a Mann-Whitney U test or Kruskal-Wallis test in the case of non-normally distributed data. For primary outcome measures, i.e. NBM volume, FA and MD of (regional) tracts, we performed a general linear model (GLM) with gender, age and educational level (dichotomized using a median split) as covariates and with a Bonferroni correction for multiple group comparisons. We used the Pearson and Spearman correlation coefficient (for normally or non-normally distributed variables, respectively) to investigate correlations between significantly altered primary outcome measures and cognitive function (CAMCOG-R total score) and general measures of disease progression, i.e. disease duration and motor performance (UPDRS III). A two-tailed *p* < 0.05 (corrected) was considered significant. All above-mentioned analyses were performed with IBM Statistical Package of the Social Sciences software version 20.0 (SPSS, Chicago, Il, USA).
